# Prediction﻿ research on sedimentation balance of Three Gorges Reservoir under new conditions of water and sediment

**DOI:** 10.1038/s41598-021-98394-x

**Published:** 2021-09-24

**Authors:** Peng Chen, Jinyun Deng, Guangming Tan, Jinyou Lu, Zhongwu Jin, Yinjun Zhou, Caiwen Shu, Zhiyong Feng, Rouxin Tang, Yiwei Lve, Yuxuan Wang

**Affiliations:** 1 Key Laboratory of River Regulation and Flood Control of Ministry of Water Resources, Changjiang River Scientific Research Institute, Wuhan, 430010 China; 2 State Key Laboratory of Water Resources and Hydropower Engineering Science, Wuhan University, Wuhan, 430072 China

**Keywords:** Hydrology, Environmental sciences

## Abstract

Influenced by climate change and human activities, especially the completion and operation of cascade reservoirs in the middle and lower reaches of Jinsha River since 2012, new changes have taken place in the water and sediment characteristics of the Three Gorges Reservoir (TGR) in recent years. In this paper, a one-dimensional unsteady water and sediment mathematical model of the main and tributary rivers of the TGR is established, and the main calculation parameters of the model are calibrated with the measured water and sediment data from January 1, 2008 to December 31, 2017. In view of the different combinations of inflow water and sediment that may occur in the TGR under the condition of new water and sediment, the long-term changes of sediment erosion and deposition and the balance of reservoir deposition in the TGR are studied using the model. The results show that: (1) Under the new conditions of water and sediment, the amount of sediment in the TGR accounts for only 14.8% and 35.8% of that in 1956–1990 and 2003–2012, respectively; (2) The variation process of water level, discharge and sediment concentration of each station along the route calculated by the model is basically consistent with the measured results, and the calculated values of total deposition amount and deposition distribution are also basically consistent with the measured results. The verification results of the model are in accordance with the measured values; (3) Under the water-sediment conditions during 1961–1970 and 1991–2000, the model predicted the estimates of 320 and 430 years for the TGR to reach a sedimentation balance, respectively. Under the new water-sediment conditions, it takes 560 years at most and 450 years at least to reach the sedimentation balance for the TGR, and the corresponding condition is the typical year with less water-less sediment and more water-more sediment, respectively. The research results of this paper can provide a new reference for the long-term safe operation and operation optimization of the TGR.

## Introduction

Affected by climatic factors (e.g., rainfall) and human activities (e.g., reservoir construction and soil and water conservation), the conditions of the sediment production and transport in the upper Yangtze River have undergone significant changes^1–7^. Without an obvious change in runoff, the sediment discharge has decreased significantly^8^. In particular, since the successive construction and operation of the cascade reservoirs (the Xiluodu and Xiangjiaba hydroelectric stations) in the middle and lower reaches of the Jinsha River in 2012, the largest cluster of reservoirs in the world has been formed in the upper Yangtze River. The completion and operation of this super cluster of reservoirs has not only changed the temporal–spatial conditions of the runoff in the basin, but it has also macroscopically changed the temporal–spatial distribution of the river sediment. Consequently, the characteristics of the water and sediment transported into the TGR has undergone new changes^9,10^.

For different areas of reservoir sedimentation research, domestic and foreign scholars also try to use different methods to do some research. Some scholars investigated debris flow deposition in the reservoir of the detention slit dams using a two-dimensional numerical model^11^; While others adopted multi-source data fusion to combine sonar sounding data, map data and manual measurement data to update and reconstruct the bottom topography of the reservoir, so as to calculate the reservoir sedimentation and its distribution^12^; Based on the artificial neural network (ANN) approach and the Modified Universal Soil Loss Equation model coupled with the multiple linear regression (MUSLE-MLR) model, Bilali et al.^13^ predicted yearly sedimentation in the Sidi Mohammed Ben Abdellah reservoir, located in a semi-arid region of Morocco. In addition, previous studies adopted non mechanism or mechanism model to study reservoir sediment transport and reservoir deposition^14–18^.

Since the 1990s, many studies have been carried out on the characteristics of water and sediment transport in the upper Yangtze River and the prediction of the volume of sediment transported into the TGR. These studies have yielded fruitful research results. Dai et al.^19^ used the statistical analysis method of the double cumulative curve of the measured annual runoff and the annual sediment discharge as well as the Spearman rank correlation test to analyze the decrease in the incoming water and sediment in the upper reaches of the TGR, and they observed correlation between the incoming water and sediment, which can be used to predict the recent annual volume of sediment transported into the TGR. Wang et al.^20^ applied the cumulative curve, the M–K sequence analysis method, and the cluster analysis method to explore the changes in the water and sediment characteristics of the trunk and tributaries in the upper Yangtze River from 1950 to 2014 and analyzed the influences of human activities. Chai et al.^21^ used the double cumulative curve to analyze the relationship between water and sediment at 7 hydrological stations in the Yangtze River Basin from 2000 to 2013. Their results show that the impoundment operation and continuous development of soil and water conservation in the TGR were the main reasons for significant decrease in the sediment discharge in the mainstream of the Yangtze River from 2000 to 2013, while the decrease in rainfall was one of the key factors leading to decline in runoff. According to the measured hydrological data after the impoundment operation of the TGR, Fu et al.^22^ revised the results of the sediment discharge ratio of the TGR reported by the Yangtze River Scientific Research Institute. Based on a series of water and sediment data from 1961 to 1970, the annual average volume of sediment transported into the TGR was predicted to decrease to 120 million t after the completion and operation of the Xiluodu, Xiangjiaba, and Jin’anqiao Reservoirs. By studying the influence of the sediment retaining effect of the reservoirs in the upper Yangtze River on the volume of sediment transported into the TGR, Li et al.^23^ concluded that on average, the sediment retaining effect of the reservoirs in the upper Yangtze River reduced the volume of sediment transported into the TGR by 110 million t/a from 1991 to 2005. Upon the operation of the proposed large reservoirs in the upper Yangtze River in the near future, the volume of sediment transported into the TGR will decrease to 90.3 million t/a, which is only 18.3% of the volume during 1956–1990. Duan et al.^24^ predicted that the volume of sediment transported into the TGR from 2015 to 2050 will decrease to about 60 million t/a, which is only about 15% of the average volume from 1991 to 2005, due to the outstanding sediment retaining effect of the reservoirs.

The above-mentioned studies provided insights into the changes in the water and sediment characteristics in the upper Yangtze River and how the sediment retaining effect of the reservoirs affects the water–sediment conditions of the TGR. Nonetheless, according to the measured data, the prediction is slightly conservative, and the sediment retaining effect of the reservoirs in the upper Yangtze River is significantly larger than expected. The volume of sediment transported into the TGR was only 34.4 million t in 2017, which indicates that the measured volume of the sediment transported into the reservoir is decreasing faster than expected. Thus, under the new water–sediment conditions, to ensure the long-term safe operation and optimized scheduling of the TGR, it is important to study the long-term scouring and silting changes and the sedimentation balance of the TGR.

In this study, based on the reservoir construction and the measured water and sediment data for the upper reaches of the Yangtze River from 1956 to 2018, the variation characteristics of the water and sediment transported into the TGR were analyzed, and the possible water–sediment combinations to the TGR in the future under new water–sediment conditions were predicted. Based on this, a one-dimensional unsteady water and sediment mathematical model of the trunk stream and tributaries of the TGR was established, and the main calculation parameters of the model were calibrated. In view of the different water–sediment combinations that may occur in the TGR under new water–sediment conditions, the model was used to study the long-term scouring and silting variations of the sediment and the sedimentation balance in the TGR area.

## Analysis of the new water–sediment conditions

The data from the Cuntan and Wulong Stations were used in the demonstration stage of the Three Gorges Project, with the sum of the annual average runoff into the reservoir being 398.6 billion m3 and the sum of the sediment discharge being 494 million t. As for the mathematical model calculation and the physical model test, the annual water and sediment data from 1961 to 1970 served as the representative water–sediment conditions, in which the annual average runoff is 420.2 billion m3 and the annual average sediment discharge is 509 million t.

With an unclear variation trend in the runoff into the reservoir, the sediment discharge has decreased. The average volume of sediment transported into the TGR was 72.5 million t from 2013 to 2018, which is only 14.8% that from 1956 to 1990 and 35.8% that from 2003 to 2012 (Table 1).

**Table 1:** Statistical data for the water and sediment characteristics from 2003 to 2018.

	Annual runoff (100 million m^3^)	Annual sediment discharge (100 million t)	Annual average sediment concentration (kg/m^3^)	Annual maximum sediment concentration (kg/m^3^)	Annual maximum water discharge (m^3^/s)	Water–sediment combinations
1956–1990	3845	4.890				
1991–2002	3733	3.510				
2003	3138	2.322	0.740	4.528	48,590	Less water and more sediment
2004	3702	1.923	0.519	4.860	59,370	
2005	4177	2.777	0.665	7.071	49,300	More water and more sediment
2006	2678	1.190	0.444	3.557	29,600	
2007	3574	2.392	0.669	5.080	44,700	Medium water and more sediment
2008	3829	2.314	0.604	3.964	36,580	
2009	3463	1.829	0.528	4.750	49,470	
2010	3721	2.291	0.616	5.227	64,060	
2011	3015	1.016	0.337	3.288	44,333	Less water and medium sediment
2012	4165	2.186	0.525	3.784	67,800	
2013	3345	1.268	0.379	15.092	46,859	Medium water and medium sediment
2014	3908	0.542	0.139	1.950	50,400	More water and less sediment
2015	3446	0.348	0.101	1.783	32,910	Less water and less sediment
2016	3805	0.417	0.109	2.859	35,310	
2017	3728	0.344	0.092	1.658	31,330	Medium water and less sediment
2018	4294	1.429	0.333	9.609	59,550	More water and medium sediment
2003–2018 (average)	3624	1.537	0.425	4.941	46,885	
2003–2012 (average)	3546	2.024				
2013–2018 (average)	3754	0.725				
Ratio 1	0.976	0.148				
Ratio 2	1.006	0.207				
Ratio 3	1.059	0.358				

Ratios 1, 2, and 3 refer to the ratios for 2013–2018 to 1956–1990, 1991–2002, and 2003–2012, respectively.

With the continuous construction of cascade reservoirs on the trunk streams and tributaries in the upper Yangtze River, the water and sediment system of the river basin has been increasingly prominently affected. The decreasing trend in the volume of sediment transported into the TGR will be faster than expected, and the rare phenomenon of a small amount of sand in a vast sea may even occur. The reduced volume of sediment transported into the reservoir will slow down the sedimentation rate of the sediment in the TGR and prolong the time required for the reservoir to reach a sedimentation balance^25^.

## Establishing the water–sediment mathematical model

### Basic equations of the model

Since there are many tributaries in the TGR area, both the water and sediment movement in the trunk stream and tributaries should be taken into consideration while establishing the one-dimensional unsteady water and sediment mathematical model of the TGR. The trunk stream and tributaries of the reservoir were regarded as single channels, and the confluence point of the rivers was defined as the branching point. The water and sediment mathematical model consists of 3 parts: the water–sediment movement equations of the single channels, the branching point connection equations, and the boundary conditions.

#### Basic equations for the water and sediment movements in a single river channel

The water flow continuity equation is^26^

1 $$\frac{{\partial A_{i} }}{\partial t} + \frac{{\partial Q_{i} }}{\partial x} = 0.$$

The water flow motion equation is

2 $$\frac{{\partial Q_{i} }}{\partial t} + \frac{\partial }{\partial x}\left( {\frac{{Q_{i}^{2} }}{{A_{i} }}} \right) + gA_{i} \left( {\frac{{\partial Z_{i} }}{\partial x} + \frac{{\left| {Q_{i} } \right|Q_{i} }}{{K_{i}^{2} }}} \right) = 0.$$

The sediment continuity equation is

3 $$\frac{{\partial Q_{i} S_{i} }}{\partial x} + \frac{{\partial A_{i} S_{i} }}{\partial t} + \alpha_{i} \omega_{i} B_{i} \left( {S_{i} - S_{*i} } \right) = 0.$$

The riverbed deformation equation is

4 $$\rho^{\prime } \frac{{\partial A_{d} }}{\partial t} = \alpha_{i} \omega_{i} B_{i} \left( {S_{i} - S_{*i} } \right).$$

here *A* is the discharge area, *Q* is the water discharge, *t* is the time, *x* refers to the coordinates along the flow path, *i* is the number of the section, *Z* is the water level, *K* is the modulus of the cross-sectional water discharge; *S* is the sediment concentration, *ρ*′ is the dry density of the sediment, *d* is the particle size, α is the recovery saturation coefficient, *ω* is the settling velocity of the sediment, *B* is the width of the cross-section, *g* is the gravitational acceleration, and *A*_*d*_ is the scouring and silting area of the riverbed.

#### Branching point connection equation

1. Discharge connection conditions

The water flowing in and out of each branching point must be balanced with the increase or decrease rate of the actual water within that branching point, that is,

5 $$\sum {Q_{i} } = \frac{\partial \Omega }{{\partial t}}$$

here $\Omega$ is the amount of water stored at the branching point. If the point is generalized as a geometric point, then $\Omega = 0$.

(2) Dynamic connection conditions

If the branching point can be generalized as a geometric point, the water flowing in and out of each branching point is gentle, and there is no sudden change in water level. Thus, the water level at the cross-section of each branching point should be the same, that is,

6 $$Z_{i} = Z_{j} = \cdots = \overline{Z} .$$

#### Boundary conditions

In the calculation, instead of giving the boundary conditions separately for a single river, the boundary conditions were determined by regarding all of the trunk stream and tributaries included in the calculation as a whole. The water discharge and sediment concentration processes were provided at the inlet of each trunk stream and tributary. The water level process, water discharge process, and the relationship between the water level and water discharge were given at the outlet of the model.

### Model solving

#### Solving the water flow equations

The third-order method was used to solve the water flow equations. First, Eqs. (1) and (2) were discretized using Pressman’s four-point implicit difference scheme, and the difference equation was obtained as follows:

7 $$B_{i1} Q_{i}^{n + 1} + B_{i2} Q_{i + 1}^{n + 1} + B_{i3} Z_{i}^{n + 1} + B_{i4} Z_{i + 1}^{n + 1} = B_{i5}$$

8 $$A_{i1} Q_{i}^{n + 1} + A_{i2} Q_{i + 1}^{n + 1} + A_{i3} Z_{i}^{n + 1} + A_{i4} Z_{i + 1}^{n + 1} = A_{i5}$$

where the coefficient was derived based on the practical conditions.

Assuming there were $mL$ cross-sections in a specific reach, the micro-segment Eqs. (7) and (8) obtained from the difference in this reach were eliminated sequentially, and then, the unknown values were concentrated at the branching point using the recursive relationship to obtain the relationship between the water level and water discharge in the cross-sections at the head and tail of this reach:

9 $$Q_{1} = \alpha_{1} + \beta_{1} Z_{1} + \delta_{1} Z_{m}$$

10 $$Q_{{m{\text{L}}}} = \theta_{{m{\text{L}}}} + \eta_{{m{\text{L}}}} Z_{1} + \gamma_{{m{\text{L}}}} Z_{{m{\text{L}}}}$$

here the coefficients $\alpha_{1}$, $\beta_{1}$, $\delta_{1}$, $\theta_{mL}$, $\eta_{mL}$, and $\gamma_{mL}$ were solved using the recursive equation.

By substituting the boundary conditions and the relationship between the water level and water discharge at the cross-sections at the head and tail of each reach into the branching point connection equation, an algebraic equation set was established with the water level at each branching point of the trunk stream and tributaries in the TGR area being unknown. The water level at each branching point was obtained by solving this equation set. Through gradual back substitution, the water discharge at the end of the reach as well as the water level and water discharge inside each reach were obtained.

#### Solving the sediment equations

The sediment continuity Eq. (3) was discretized using the explicit scheme:

11 $$S_{i}^{j + 1} = \frac{{\Delta t\alpha_{i}^{j + 1} B_{i}^{j + 1} \omega_{i}^{j + 1} S_{ * i}^{j + 1} + A_{i}^{j} S_{i}^{j} + \frac{\Delta t}{{\Delta x_{i - 1} }}Q_{i - 1}^{j + 1} S_{i - 1}^{j + 1} }}{{A_{i}^{j + 1} + \Delta t\alpha_{i}^{j + 1} B_{i}^{j + 1} \omega_{i}^{j + 1} + \frac{\Delta t}{{\Delta x_{i - 1} }}Q_{i}^{j + 1} }}.$$

By substituting Eq. (3) into Eq. (4), the riverbed deformation Eq. (4) was discretized to obtain

12 $$\Delta A_{di} = \frac{{\Delta t(Q_{i - 1}^{j + 1} S_{i - 1}^{j + 1} - Q_{i}^{j + 1} S_{i}^{j + 1} )}}{{\Delta x\rho^{\prime } }} + \frac{{A_{i}^{j} S_{i}^{j} - A_{i}^{j + 1} S_{i}^{j + 1} }}{{\rho^{\prime } }}$$

here $\Delta x$ is the spatial step; $\Delta t$ is the time step; $\Delta A_{di}$ is the deformation area of the riverbed with suspended sediment; and corner mark *j* is the time layer.

Once the water level and water discharge in all of the cross-sections of the trunk stream and tributaries were calculated, the sediment concentration of each cross-section was solved from top to bottom using Eq. (11). The sediment distribution ratio at the branching point was set as equal to the diversion ratio, and the riverbed deformation was calculated using Eq. (12).

## Verification of the mathematical model

### Verification of the computational conditions

#### Basic conditions

**Figure 1:**
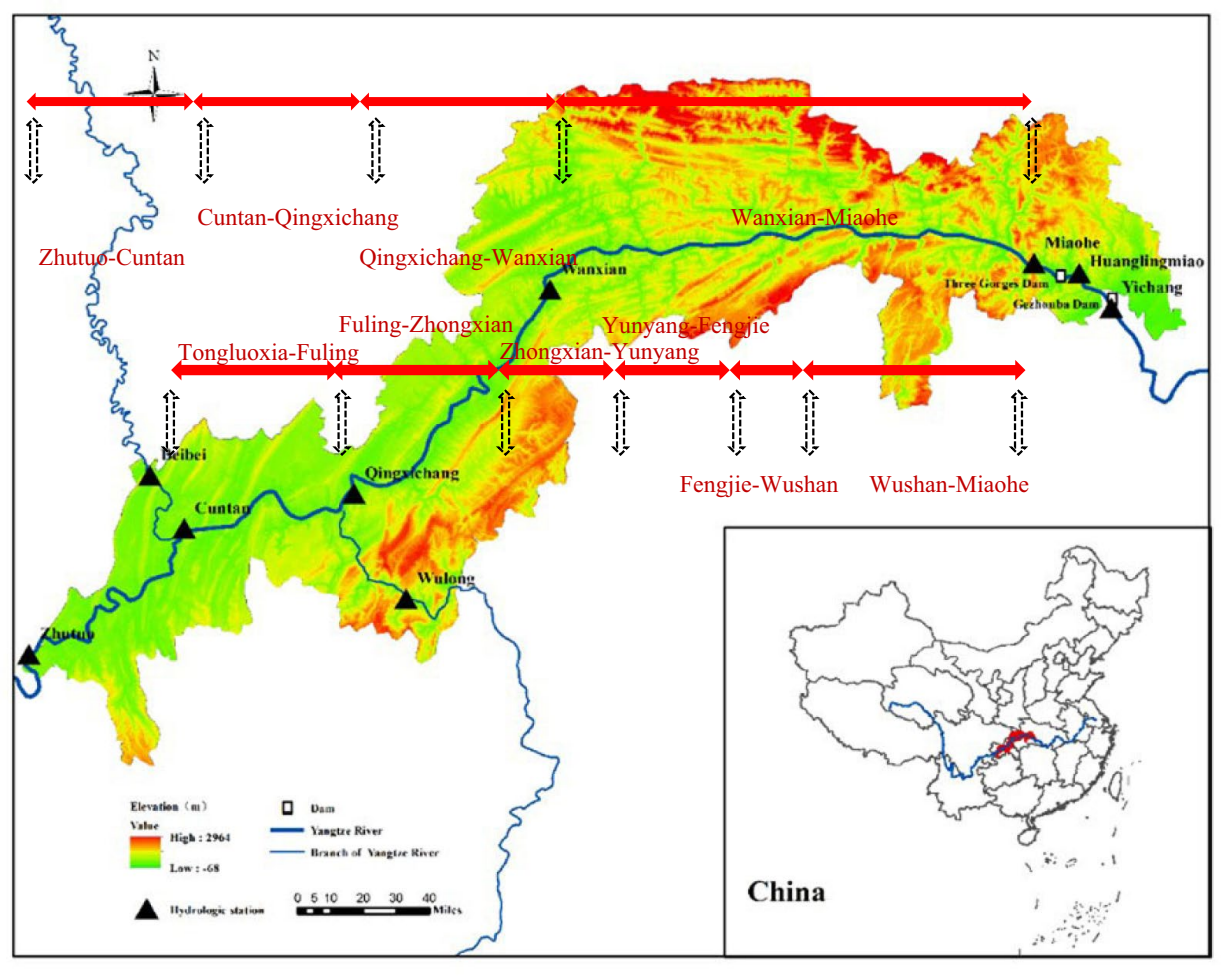
Sketch map of the TGR area.

1. The sediment used in the calculation was non-uniform. The sediment was divided into 10 groups based on particle sizes, i.e., 0.002 mm, 0.002–0.004 mm, 0.004–0.008 mm, 0.008–0.016 mm, 0.016–0.031 mm, 0.031–0.062 mm, 0.062–0.125 mm, 0.125–0.250 mm, 0.250–0.50 mm, and 0.5–1 mm. The riverbed was divided into 3 layers: the surface layer, the middle layer, and the bottom layer. The surface layer is the sediment exchange layer, the middle layer is the transition layer, and the bottom layer is the limit layer of the sediment scouring.
2. The water–sediment conditions at the inlet and outlet were calculated. The average daily water discharge and sediment concentration at Zhutuo Station on the trunk stream, Beibei Station on the Jialing River, and Wulong Station on the Wujiang River from January 1, 2008, to December 31, 2017, were used to calculate the water–sediment conditions at the inlet, with the monthly average gradation being the granular composition of the suspended sediment.
3. The scope of the reach was calculated. Stretching from Zhutuo to Daba in the TGR area (Fig. 1), the calculated reach includes the trunk stream of the Yangtze River and some of the segments of the Jialing River and Wujiang River (tributaries of the Yangtze River). With a length of about 400 km, the trunk stream was divided into 400 calculated sections, with each section being an average of 1.9 km long. The 61.3 km long Jialing River flowing from Beibei to the estuary was divided into 26 sections, with each section being an average of 2.4 km long. The Wujiang River, which is 67.7 km long and flows from Wulong to the estuary, was divided into 36 sections, of which each is an average of 1.9 km long.

#### Calibration of the roughness coefficient

The roughness coefficient was calibrated based on the relationship between the water level and the water discharge measured at each station before the impoundment and during the initial impoundment stage of the TGR. The entire reach of the trunk stream was divided into several segments. The roughness coefficients of the Jialing River and the Wujiang River were assigned based on experience, and the calibration results are shown in Table 2.

**Table 2:** Statistical table of the piecewise comprehensive roughness coefficients of the TGR.

Segment	Item	Roughness coefficient corresponding to different water discharge						
**Trunk stream of the Yangtze River**								
Zhutuo-Cuntan	Water discharge	2000	5000	10,000	20,000	40,000	70,000	
	Roughness coefficient	0.07	0.05	0.04	0.036	0.035	0.033	
Cuntan-Qingxichang	Water discharge	2000	5000	10,000	20,000	30,000	70,000	
	Roughness coefficient	0.045	0.045	0.045	0.046	0.047	0.047	
Qingxichang-Zhongxian	Water discharge	2000	5000	10,000	20,000	30,000	40,000	70,000
	Roughness coefficient	0.031	0.035	0.038	0.042	0.044	0.044	0.044
Zhongxian-Wanzhou	Water discharge	1000	3000	5000	10,000	20,000	40,000	70,000
	Roughness coefficient	0.045	0.042	0.041	0.040	0.045	0.045	0.045
Wanzhou-Fengjie	Water discharge	2000	5000	10,000	20,000	30,000	40,000	70,000
	Roughness coefficient	0.044	0.041	0.042	0.05	0.06	0.06	0.06
Fengjie-the dam site	Water discharge	2000	5000	10,000	20,000	30,000	40,000	70,000
	Roughness coefficient	0.048	0.048	0.05	0.055	0.068	0.073	0.073
**Jialing River**								
Beibei-Huikou	Water discharge	500	1000	2000	5000	10,000	20,000	30,000
	Roughness coefficient	0.02	0.02	0.032	0.042	0.048	0.053	0.053
**Wujiang River**								
Wulong-Huikou	Water discharge	100	500	1000	2000	5000	10,000	
	Roughness coefficient	0.065	0.06	0.055	0.05	0.05	0.05	

It is difficult to directly determine the roughness coefficient beyond the wet perimeter of the natural flood water level, and since the granular composition of bed sediments changes in the process of reservoir sedimentation, it is relatively difficult to determine the change in the roughness coefficient during the process of reservoir sedimentation. In this study, the proposed model suggests that the bed sand resistance and the sidewall resistance were assumed to determine the variation in the roughness coefficient during sedimentation.

### Operation of the TGR during the verification

**Figure 2:**
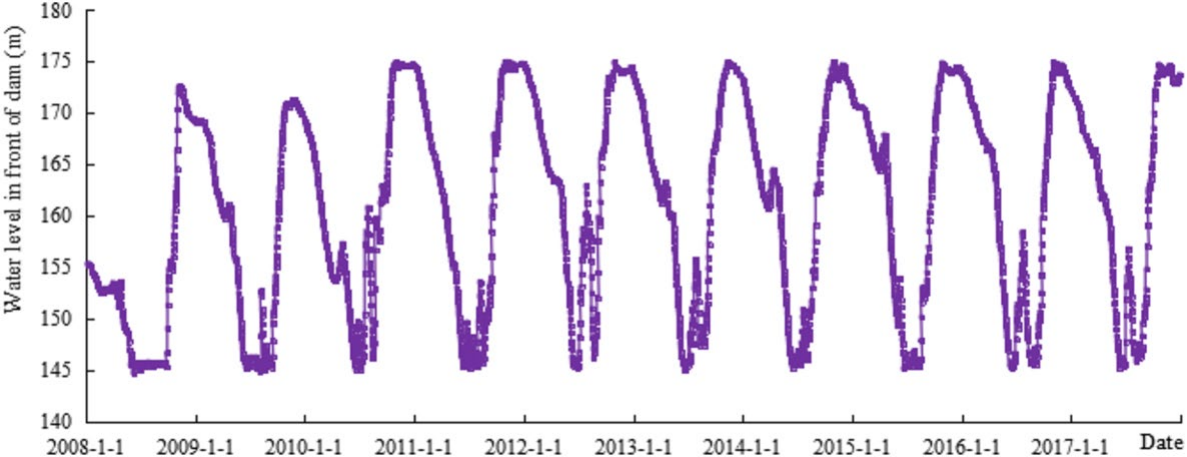
Water level process in front of the TGR from 2008.1.1 to 2017.12.31.

The verification stage in this study lasted from January 1, 2008, to December 31, 2017. The reservoir operated with the water level in front of the dam following the initial operation mode from January 2008 to September 2008; and from October 2008 to December 2017, and the reservoir operated with water levels in front of the dam of 175–145–155 m (Fig. 2).

### Verification of the water level, water discharge, and sediment concentration processes

**Figure 3:**
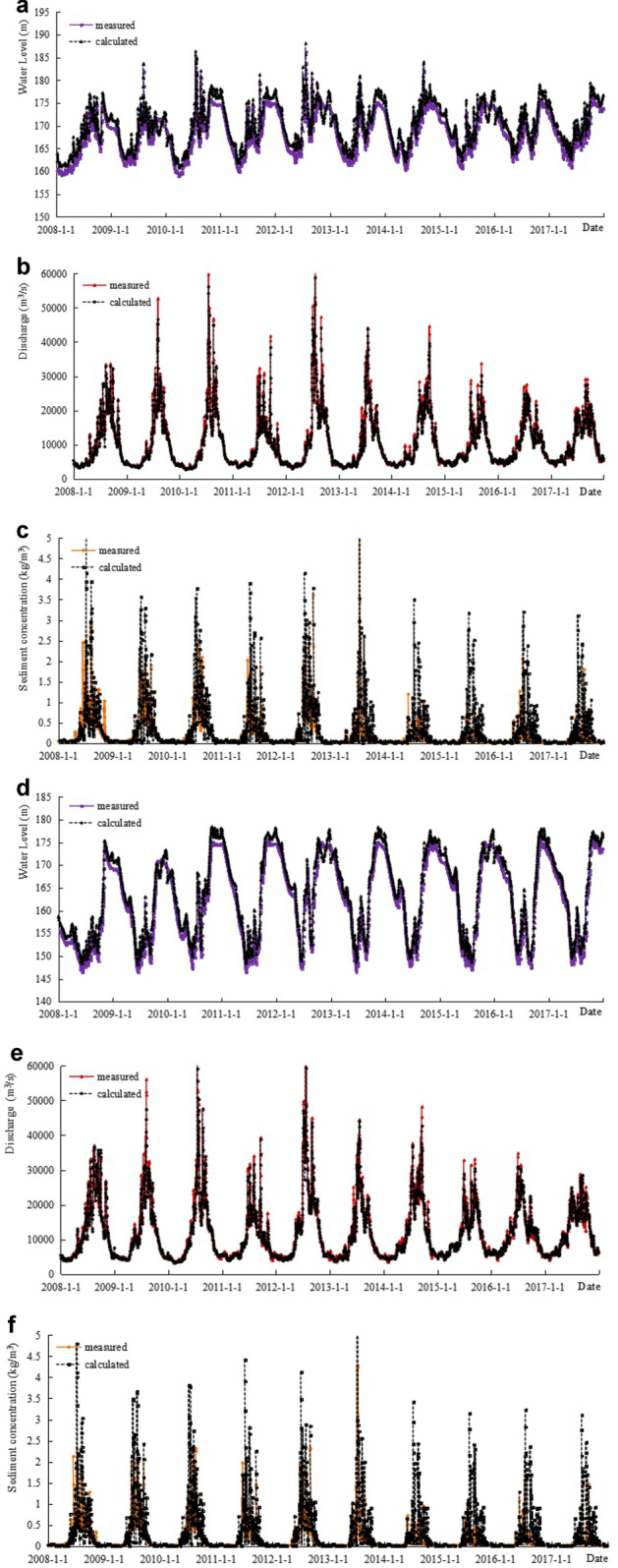

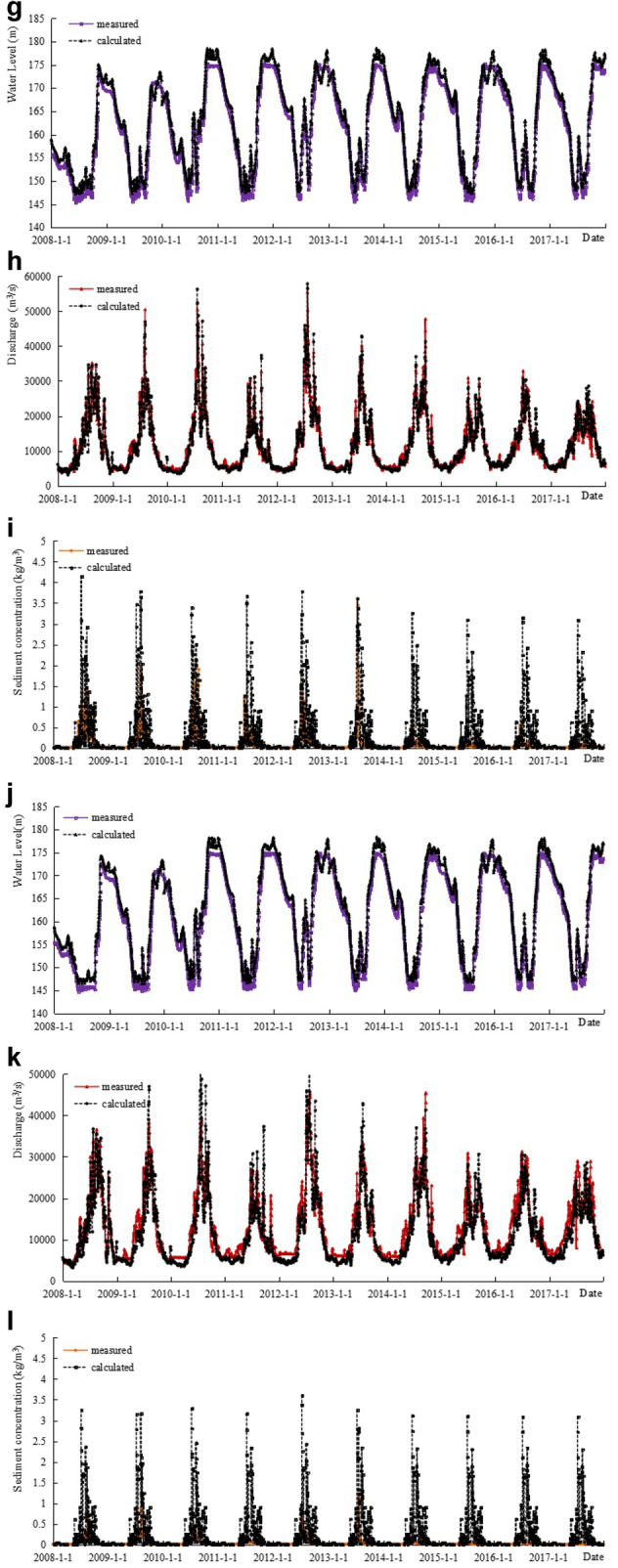
**a** Verification results of water level at Cuntan from 2008.1.1 to 2017.12.31. **b** Verification results of discharge at Cuntan from 2008.1.1 to 2017.12.31. **c** Verification results of sediment concentration at Cuntan from 2008.1.1 to 2017.12.31. **d** Verification results of water level at Qingxichang from 2008.1.1 to 2017.12.31. **e** Verification results of discharge at Qingxichang from 2008.1.1 to 2017.12.31. **f** Verification results of sediment concentration at Qingxichang from 2008.1.1 to 2017.12.31. **g** Verification results of water level at Wanxian from 2008.1.1 to 2017.12.31. **h** Verification results of discharge at Wanxian from 2008.1.1 to 2017.12.31. **i** Verification results of sediment concentration at Wanxian from 2008.1.1 to 2017.12.31. **j** Verification results of water level at Miaohe from 2008.1.1 to 2017.12.31. **k** Verification results of discharge at Miaohe from 2008.1.1 to 2017.12.31. **l** Verification results of sediment concentration at Miaohe from 2008.1.1 to 2017.12.31.

Based on currently available measured data, the calculated results were compared with the water level, water discharge, and sediment concentration process measured at the major hydrological stations along the route from January 1, 2008, to December 31, 2017. The results are shown in Fig. 3a–l. As shown in the figures, the variations in the water level, water discharge, and sediment concentration calculated by the model are consistent with that of the measured results, and the calculated results verified by the model are in good agreement with the measured values.

Based on the analysis of the error range of the daily flow process from January 1, 2008 to December 31, 2017, the water level error of each station within the ± 0.4 m interval has a confidence of about 80%, the water discharge error of each station within the ± 10% interval has a confidence coefficient of about 90%, and the sediment concentration error of each station within the ± 30% interval has a confidence of approximately 80% (Table 3).

**Table 3:** Analysis of the verification error range of the daily water flow process in the TGR from January 1, 2008, to December 31, 2017.

Station	Water level		Water discharge		Sediment concentration	
	Error range (m)	Confidence (%)	Error range (%)	Confidence (%)	Error range (%)	Confidence (%)
Cuntan	± 0.1	39	± 2	46	± 10	53
	± 0.2	61	± 5	85	± 20	74
	± 0.4	82	± 10	98	± 30	90
Qingxichang	± 0.1	23	± 2	31	± 10	35
	± 0.2	54	± 5	60	± 20	52
	± 0.4	75	± 10	88	± 30	80
Wanxian	± 0.1	36	± 2	24	± 10	35
	± 0.2	62	± 5	59	± 20	56
	± 0.4	81	± 10	85	± 30	72
Miaohe	± 0.1	39	± 2	26	± 10	38
	± 0.2	61	± 5	58	± 20	54
	± 0.4	83	± 10	87	± 30	75

### Verification of the sediment discharge

Based on the verification results of the sediment concentration process of several major hydrological stations in the TGR area shown in Fig. 3 from January 1, 2008 to December 31, 2017, the calculated results of each station are basically consistent with the measured values, but the peak value of the calculated sediment concentration is smaller than the measured value. However, the calculated value is slightly larger for the medium and small water discharges. Since the calculated value is close to the measured value during most periods of time, the cumulative sediment discharge throughout the year is relatively close to the measured value (Table 4).

**Table 4:** Verification table of the sediment discharge at the main stations (unit: 100 million t).

Time period (year)	Cuntan		Qingxichang		Wanxian		Miaohe	
	Measured	Calculated	Measured	Calculated	Measured	Calculated	Measured	Calculated
2008	2.126	2.315	1.893	2.320	1.051	1.209	0.388	0.588
2009	1.733	1.993	1.824	2.099	1.055	1.206	0.433	0.518
2010	2.111	2.231	1.942	2.267	1.150	1.246	0.327	0.534
2011	0.916	1.178	0.883	1.275	0.309	0.533	0.077	0.224
2012	2.105	2.238	1.902	2.229	1.144	1.246	0.459	0.625
2013	1.207	1.416	1.206	1.439	0.849	0.964	0.372	0.404
2014	0.519	0.863	0.561	0.992	0.234	0.579	0.131	0.382
2015	0.328	0.585	0.352	0.669	0.113	0.375	0.041	0.259
2016	0.425	0.484	0.413	0.556	0.197	0.398	0.082	0.260
2017	0.347	0.663	0.304	0.741	0.108	0.343	0.043	0.276
Total	11.818	13.966	11.280	14.587	6.210	8.098	2.352	4.068

### Comprehensive verification of the sedimentation volume

#### Sedimentation volume and its distribution (based on the cross-section)

According to the report^27^ issued by the Hydrology Bureau of the Changjiang Water Resources Commission in March 2018, from 2008 to 2017, the measured cumulative sedimentation volume in the trunk stream of the reservoir area was 850.8 million m^3^ (Table 5), of which the measured scoured quantity in the variable backwater area (Tongluoxia-Fuling) is 24.9 million m^3^, and the measured sedimentation volumes in the perennial backwater area (Miaohe-the dam site, Wushan-Miaohe, Fengjie-Wushan, Yunyang-Fengjie, Zhongxian-Yunyang, and Fuling-Zhongxian) are 65.9 million m^3^, 98.5 million m^3^, 26.4 million m^3^, 64.2 million m^3^, 412.3 million m^3^, and 206.3 million m^3^, respectively. According to the calculation results, the calculated value for the trunk stream of the reservoir area (Tongluoxia-the dam site) is 928.2 million m^3^, which is 79 million m^3^ larger than the measured value, with the relative error being 9.3%. The calculated value in the variable backwater area (Tongluoxia-Fuling) is − 26.4 million m^3^, with a relative error of 6.2%. Except for the relatively large error between the calculated and measured values in the river segment from Miaohe to the dam site, the relative error between the calculated and measured values of the other river segments in the perennial backwater area was less than 10%. According to the verification of the sedimentation volume of typical reaches of the trunk stream in the TGR area (Table 6), the measured sedimentation volumes of the Huanghuacheng segment, the Lanzhuba segment, the Fengweiba segment, the Tunaozi segment, the Qingyanzi segment, and the Luoqi segment were 60.548 million m^3^, 32.631 million m^3^, 20.089 million m^3^, 11.683 million m^3^, − 12.7055 million m^3^, and − 14.486 million m^3^, respectively. Compared to the measured results, except for the relatively large error of the sedimentation volume of the Qingyanzi segment, the relative errors between the calculated and measured values of the other typical river segments were all less than 5%. Thus, the calculated cumulative sedimentation volume and the sedimentation distribution are basically consistent to the measured values.

**Table 5:** Verification of the sedimentation volume and distribution in the trunk stream in the TGR area from 2008 to 2017 (based on the cross-section).

Segment	River length (km)	Measured (100 million m^3^)	Calculated	Absolute error (100 million m^3^)	Relative error (%)
Tongluoxi-Fuling	111.4	− 0.249	− 0.264	− 0.015	6.2
Fuling-Zhongxian	113.9	2.063	2.242	0.179	8.7
Zhongxian-Yunyang	66.7	4.123	4.292	0.169	4.1
Yunyang-Fengjie	67.8	0.642	0.704	0.062	9.7
Fengjie-Wushan	35.5	0.264	0.252	− 0.012	− 4.5
Wushan-Miaohe	106.3	0.985	0.895	− 0.091	− 9.2
Miaohe-Daba	15.1	0.659	0.966	0.307	46.5
Tongluoxia-Daba	597.9	8.492	9.282	0.790	9.3

**Table 6:** Verification of the sedimentation volume in typical reaches of the trunk stream in the TGR area from 2008 to 2017 (based on the cross-section).

Segment	River length (km)	Measured (10^4^ m^3^)	Calculated (10^4^ m^3^)	Absolute error (10^4^ m^3^)	Relative error (%)
Luoqi segment	30.0	− 1448.6	− 1509.441	− 60.841	4.2
Qingyanzi segment	15.0	− 1270.55	− 1350.595	− 80.045	6.3
Tunaozi segment	3.0	1168.3	1220.874	52.574	4.5
Fengweiba segment	5.46	2008.9	2105.327	96.427	4.8
Lanzhuba segment	6.08	3263.1	3181.523	− 81.578	− 2.5
Huanghuacheng segment	5.1	6054.8	6169.841	115.041	1.9

#### Sedimentation volume and its process (based on the sediment discharge)

From June 1, 2003, when the TGR began operating, to 2017, the reservoir was characterized by overall sedimentation. The river segment from Zhutuo to the dam site were selected as the river segment for sedimentation statistical analysis in this study. Based on the calculation performed using the sediment discharge method, the measured cumulative sedimentation volume in the reservoir area is 1.029 billion t (Table 7) and the calculated value is 1.065 billion t, which is 36 million t larger than the measured value, with a relative error of 3.5%. Judging from the sedimentation process, the error between the calculated and measured values is comparatively small, except for that in 2013. In addition, the absolute difference between the measured and calculated sedimentation volumes is less than 10 million t, with the relative error being less than 10.9%. Therefore, the cumulative sedimentation volume and process calculated using the model are in good agreement with the measured results.

**Table 7:** Verification of the annual sedimentation volume process after impoundment in the TGR (based on the water discharge).

Time period (year)	Measured (100 million t)	Calculated (100 million t)	Absolute error (100 million t)	Relative error (%)
2008	1.856	1.828	− 0.028	− 1.5
2009	1.470	1.523	0.053	3.6
2010	1.962	1.986	0.024	1.2
2011	0.951	0.980	0.029	3.1
2012	1.737	1.803	0.066	3.8
2013	0.942	1.045	0.103	10.9
2014	0.449	0.493	0.044	9.8
2015	0.278	0.299	0.021	7.6
2016	0.334	0.355	0.021	6.4
2017	0.312	0.339	0.027	8.7
Total	10.290	10.650	0.360	3.5

## Application of the mathematical model

### Setting of the computational schemes

Affected by climate change and human activities, especially the gradual construction of cascade power stations in the lower reaches of the Jinsha River, the impoundment and sediment retaining effect of the cascade reservoirs in the basin have begun to exert a new influence on the characteristics of the water and sediment transported into the TGR. According to the measured data, the sediment retaining effect of the reservoirs in the upper Yangtze River was stronger than expected. From 2013 to 2018, the annual average volume of sediment transported into the TGR is 72.5 million t, which is only 14.8% that from 1956 to 1990 and 35.8% that from 2003 to 2012. The volume of sediment transported into the reservoir in 2017 was only 34.4 million t, indicating that the measured volume of sediment transported into the reservoir is decreasing faster than expected. In view of the new water–sediment conditions, in this study, the sedimentation volume in the TGR was calculated over a long timespan, i.e., 600 years. Based on the measured annual runoff and sediment discharge from 2003 to 2018, the water–sediment combinations were determined as follows: the year with less water-more sediment (2003), the year with more water-more sediment (2005), the year with medium amount of water-more sediment (2007), the year with less water-medium amount of sediment (2011), the year with medium amount of water-medium amount of sediment (2013), the year with more water-less sediment (2014), the year with less water-less sediment (2015), the year with medium amount of water-less sediment (2017), and the year with more water-medium amount of sediment (2018). Moreover, the water–sediment combination during 1961–1970 and that during 1991–2000 were selected as the basic schemes for comparison.

The actual scheduling scheme of the TGR after impoundment served as the scheduling scheme in this study. After October 2008, the reservoir entered the experimental impoundment stage, with the follow-up scheduling schemes being consistent. In other words, the water level in front of the dam was set as 135–139 m from June 2003 to June 2006; the reservoir operated with the water level in front of the dam being 144–156 m from September 2006 to September 2008; and the water level in front of the dam was set as 175–145–155 m from October 2008 to June 2602 (Table 8).

**Table 8:** Computational conditions of the different schemes.

Scheme No	Name	Water–sediment conditions	Water level in front of the dam
1	Basic scheme	Water–sediment conditions in 1961–1970	The reservoir operated with the water level in front of the dam being 135–139 m from June 2003 to June 2006; the reservoir operated with the water level in front of the dam being 144–156 m from September 2006 to September 2008; the reservoir operated with the water level in front of the dam being 175–145–155 m from October 2008 to June 2602
2		Water–sediment conditions in 1991–2000	
3	Smaller water and larger sediment	Water–sediment conditions in 2003	The same as above
4	Larger water and larger sediment	Water–sediment conditions in 2005	The same as above
5	Medium water and larger sediment	Water–sediment conditions in 2007	The same as above
6	Smaller water and medium sediment	Water–sediment conditions in 2011	The same as above
7	Medium water and medium sediment	Water–sediment conditions in 2013	The same as above
8	Larger water and smaller sediment	Water–sediment conditions in 2014	The same as above
9	Smaller water and smaller sediment	Water–sediment conditions in 2015	The same as above
10	Medium water and smaller sediment	Water–sediment conditions in 2017	The same as above
11	Larger water and medium sediment	Water–sediment conditions in 2018	The same as above

### Results and discussions

With the development of reservoir sedimentation, the reservoir finally entered a phase of sedimentation balance. According to the shape of the riverbed, the sedimentation balance can be divided into the longitudinal balance and the horizontal balance, and the sediment transport can be classified into the suspended sediment balance and the bed load balance^28^. Since this study focuses on the suspended sediment balance, when the sedimentation balance were along the longitudinal profile of the reservoir, it was considered that the reservoir sedimentation has basically reached a balanced state.

**Figure 4:**
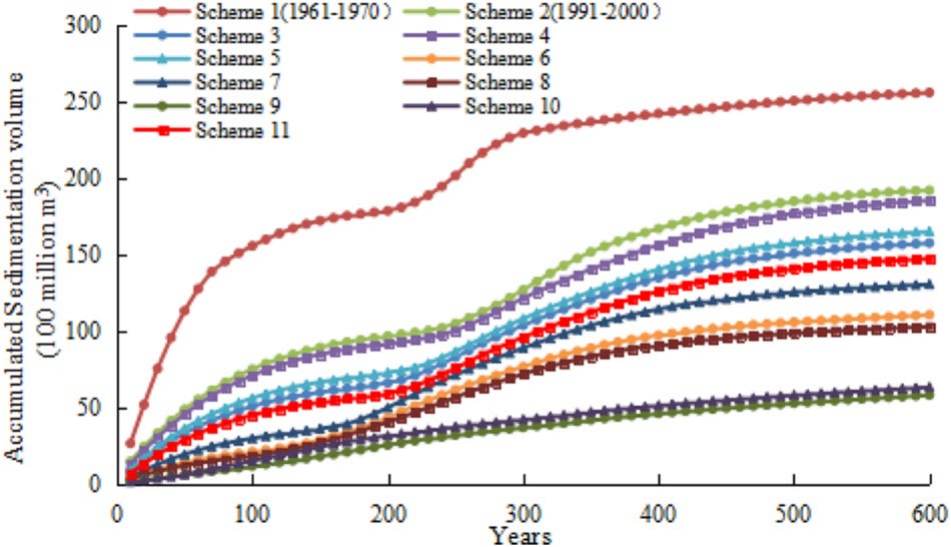
Accumulation process of the TGR under different calculation schemes.

As shown in Fig. 4, 2 periods of time, i.e., 1961–1970 and 1991–2000, were involved in the calculation of the basic schemes. As for Scheme 1, under the water–sediment conditions in 1961–1970, after 100 years of operation, the cumulative sedimentation volume was 15.569 billion m^3^ in the reservoir. In Scheme 2, under the water–sediment conditions in 1991–2000, the cumulative sedimentation volume was 7.499 billion m^3^ after 100 years of operation, which was 8.07 billion m^3^ less than that of scheme 1.

Under the water–sediment conditions of scheme 1, the cumulative sedimentation volumes were 15.569 billion m^3^, 22.96 billion m^3^, and 25.077 billion m^3^ after 100, 300, and 500 years of reservoir operation respectively, with the reservoir being in a state of cumulative sedimentation at this stage. From the perspective of the sedimentation rate, the sedimentation was rapid during the first 100 years, decreased from the 100 to the 210 years, and followed by an increasing trend. After 320 years of operation, the sedimentation declined again, and there was an inflection point on the cumulative sedimentation hydrograph. The cumulative sedimentation volume was 23.288 billion m^3^ after 320 years of operation. Thus, the reservoir sedimentation is basically balanced.

Under the water–sediment conditions of scheme 2, the cumulative sedimentation volumes were 7.499 billion m^3^, 12.69 billion m^3^, and 18.5 billion m^3^ after 100, 300, and 500 years of reservoir operation respectively, with the reservoir being in a state of cumulative sedimentation at this stage. From the perspective of the sedimentation rate, the sedimentation was comparatively fast during the first 150 years, decreased from the 150 to the 240 years, and followed by an increasing trend. After 430 years of operation, the sedimentation declined again, and there was an inflection point on the cumulative sedimentation hydrograph. The cumulative sedimentation volume was 17.43 billion m^3^ after 430 years of operation. Thus, the reservoir sedimentation is basically balanced.

Under the water–sediment conditions of scheme 3, the cumulative sedimentation volumes were 5.068 billion m^3^, 10.37 billion m^3^, 15.086 billion m^3^, and 15.763 billion m^3^ after 100, 300, 500, and 600 years of reservoir operation respectively, with the reservoir being in a state of cumulative sedimentation at this stage. From the perspective of the sedimentation rate, the sedimentation was relatively fast during the first 140 years, decreased from the 140 to the 210 years, and followed by an increasing trend. After 480 years of operation, the sedimentation declined again, and there was an inflection point on the cumulative sedimentation hydrograph. The reservoir sedimentation experiences a basic balance, and the cumulative sedimentation volume was 14.869 billion m^3^ after 480 years of operation.

Under the water–sediment conditions of scheme 4, the cumulative sedimentation volumes were 7.092 billion m^3^, 12.148 billion m^3^, 17.706 billion m^3^, and 18.567 billion m^3^ after 100, 300, 500, and 600 years of reservoir operation respectively, with the reservoir being in a state of cumulative sedimentation at this stage. Regarding the sedimentation rate, the sedimentation was relatively fast during the first 160 years, followed by a declining trend from the 160 to the 240 years, and increased after 240 years. After 450 years of operation, the sedimentation declined again, and there was an inflection point on the cumulative sedimentation hydrograph. The cumulative sedimentation volume was 16.87 billion m^3^ after 450 years of operation. Thus, the reservoir sedimentation is basically balanced.

Under the water–sediment conditions of scheme 5, the cumulative sedimentation volumes were 5.601 billion m^3^, 10.802 billion m^3^, 15.79 billion m^3^, and 16.526 billion m^3^ after 100, 300, 500, and 600 years of operation reservoir respectively, with the reservoir being in a state of cumulative sedimentation. From the perspective of the sedimentation rate, the sedimentation was relatively fast during the first 130 years, decreased from the 130 to the 240 years, and followed by an increasing trend. After 470 years of operation, the sedimentation declined again, and there was an inflection point on the cumulative sedimentation hydrograph. The cumulative sedimentation volume was 15.428 billion m^3^ after 470 years of operation. Thus, the reservoir sedimentation is basically balanced.

Under the water–sediment conditions of scheme 6, the cumulative sedimentation volumes were 2.107 billion m^3^, 7.661 billion m^3^, 10.581 billion m^3^, and 11.059 billion m^3^ after 100, 300, 500, and 600 years of reservoir operation respectively, with the reservoir being in a state of cumulative sedimentation. From the perspective of the sedimentation rate, the sedimentation was comparatively fast during the first 80 years, decreased from the 80 to the 140 years, and followed by an increasing trend. After 520 years of operation, the sedimentation declined again, and there was an inflection point on the cumulative sedimentation hydrograph. The reservoir sedimentation enters a basically balanced state, and the cumulative sedimentation volume was 10.684 billion m^3^ after 520 years of operation.

Under the water–sediment conditions of scheme 7, the cumulative sedimentation volumes were 2.971 billion m^3^, 8.888 billion m^3^, 12.561 billion m^3^, and 13.062 billion m^3^ after 100, 300, 500, and 600 years of reservoir operation respectively, with the reservoir being in a state of cumulative sedimentation. From the perspective of the sedimentation rate, the sedimentation was relatively fast during the first 110 years, decreased from the 110 to the 170 years, and followed by an increasing trend. After 510 years of operation, the sedimentation declined again, and there was an inflection point on the cumulative sedimentation hydrograph. The cumulative sedimentation volume was 12.624 billion m^3^ after 510 years of operation. Thus, the reservoir sedimentation is basically balanced.

Under the water–sediment conditions of scheme 8, the cumulative sedimentation volumes were 1.847 billion m^3^, 7.179 billion m^3^, 9.877 billion m^3^, and 10.293 billion m^3^ after 100, 300, 500, and 600 years of reservoir operation respectively, with the reservoir being in a state of cumulative sedimentation. From the perspective of the sedimentation rate, the sedimentation was comparatively fast during the first 90 years, decreased from the 90 to the 140 years, and followed by an increasing trend. After 530 years of operation, the sedimentation declined again, and there was an inflection point on the cumulative sedimentation hydrograph. The reservoir sedimentation enters a basically balanced state, and the cumulative sedimentation volume was 10.021 billion m^3^ after 530 years of operation.

Under the water–sediment conditions of scheme 9, the cumulative sedimentation volumes were 1.154 billion m^3^, 3.689 billion m^3^, 5.275 billion m^3^, and 5.806 billion m^3^ after 100, 300, 500, and 600 years of reservoir operation respectively, with the reservoir being in a state of cumulative sedimentation. From the perspective of the sedimentation rate, the cumulative sedimentation rate is relatively stable, exhibiting a slow and uniform sedimentation trend. After 560 years of operation, there was an inflection point on the cumulative sedimentation hydrograph. The cumulative sedimentation volume was 5.607 billion m^3^ after 560 years of operation. Thus, the reservoir sedimentation is basically balanced.

Under the water–sediment conditions of scheme 10, the cumulative sedimentation volumes in the reservoir area were 1.497 billion m^3^, 4.215 billion m^3^, 5.803 billion m^3^, and 6.328 billion m^3^ after 100, 300, 500, and 600 years of reservoir operation respectively, with the reservoir being in a state of cumulative sedimentation. From the perspective of the sedimentation rate, the cumulative sedimentation rate was relatively steady, exhibiting a slow and uniform sedimentation trend. After 540 years of operation, there was an inflection point on the cumulative sedimentation hydrograph. The reservoir sedimentation was a basically balanced state, and the cumulative sedimentation volume was 6.03 billion m^3^ after 540 years of operation.

Under the water–sediment conditions of scheme 11, the cumulative sedimentation volumes were 4.491 billion m^3^, 9.588 billion m^3^, 14.105 billion m^3^, and 14.748 billion m^3^ after 100, 300, 500, and 600 years of reservoir operation respectively, with the reservoir being in a state of cumulative sedimentation. From the perspective of the sedimentation rate, the sedimentation was comparatively fast during the first 130 years, decreased from the 130 to the 210 years, and followed by an increasing trend. After 500 years of operation, the sedimentation declined again, and there was an inflection point on the cumulative sedimentation hydrograph. The reservoir sedimentation enters a basically balanced state, and the cumulative sedimentation volume was 14.105 billion m^3^ after 500 years of operation.

In summary, under the water–sediment conditions during 1961–1970 and 1991–2000, it takes 320 years and 430 years, respectively, for the TGR to reach a sedimentation balance. Under the new water–sediment conditions, it takes 560 years at most and 450 years at least for the TGR to reach a sedimentation balance, with the corresponding water–sediment conditions being a typical year with less water-less sediment and a typical year with more water-more sediment, respectively.

As shown in Table 9, it takes 320 years and 430 years respectively for the TGR to reach the sediment balance under the water–sediment conditions during 1961–1970 and 1991–2000. Affected by climate change and human activities, especially the construction and operation of cascade hydropower stations on the Jinsha River in the upper Yangtze River, the conditions of the water and sediment transported into the TGR have experienced new changes. Under the new water–sediment conditions, it takes 560 years at most and 450 years at least for the TGR to reach a sedimentation balance, with the corresponding water–sediment conditions being a typical year with a small water flow and a small amount of sediment and a typical year with less water-less sediment and a typical year with more water-more sediment, respectively.

**Table 9:** Time required by the different computational schemes to achieve sedimentation balance.

Computational conditions	Annual runoff (100 million m^3^)	Annual sediment discharge (100 million t)	Annual maximum sediment concentration (kg/m^3^)	Annual maximum water discharge (m^3^/s)	Water–sediment combinations	Time required to achieve sedimentation balance (years)
1961–1970	3976	5.418			Water–Sediment conditions in 1961–1970	320
1991–2000	3754	3.682			Water–Sediment conditions in 1991–2000	430
2003	3138	2.322	4.528	48,590	Small water flow and large sediment	480
2005	4177	2.777	7.071	49,300	Large water flow and large sediment	450
2007	3574	2.392	5.080	44,700	Medium water flow and large sediment	470
2011	3015	1.016	3.288	44,333	Small water flow and medium sediment	520
2013	3345	1.268	15.092	46,859	Medium water flow and medium sediment	510
2014	3908	0.542	1.950	50,400	Large water flow and small sediment	530
2015	3446	0.348	1.783	32,910	Small water flow and small sediment	560
2017	3728	0.344	1.658	31,330	Medium water flow and small sediment	540
2018	4294	1.429	9.609	59,550	Large water flow and medium sediment	500

Many studies have been locally and globally conducted on reservoir sedimentation prediction. Maris et al.^29^ predicted the sedimentation area and the sediment deposition height in the Nipsa reservoir using a Geographical Information System (GIS) based TopRunDF model. As a result, the model predicted a significant future decrease in the reservoir stored water volume. However, the model can only predict the inflow runoff of the reservoir, not the sediment deposition of the reservoir. Using bathymetric survey, Ethiopian scholars^30^ studied the deposition of the abrajit reservoir in the North gojem sub basin of the Blue Nile River Basin, and estimated the water storage limit of the dam. However, this method only provides an estimate, and it is difficult to accurately estimate the limit of reservoir sedimentation balance. Abebe Tadesse et al.^31^ used the Soil and Water Assessment Tool(SWAT) model and Hydrologic Engineering Center-River Analysis System(HEC-RAS) model to estimate the sediment load reaching the Koka Dam Reservoir in Ethiopia. Although the model can predict the total amount of reservoir sedimentation, it does not explain how to reach the equilibrium of reservoir sedimentation under different water and sediment conditions. Taking into account the Longpan Cascade Hydropower Station, Huang^32^ reported that the reservoir reached a sedimentation balance after approximately 340 years of operation. Without considering the Longpan Cascade Hydropower Station, however, the model predicted an estimate of 370 years for the reservoir to reach a sedimentation balance based on the water–sediment conditions during 1991–2000. Because of the different initial conditions, boundary conditions, and scheduling modes, the calculation results are different under the two different water–sediment conditions. Nevertheless, the results of this study are in accordance with the measured results, which confirms the rationality and reliability of the findings of our study.

## Conclusions

1. Under the new water–sediment conditions, the volume of sediment transported into the TGR accounted for only 14.8% and 35.8% of the amount of water and sediment transported into the reservoir during 1956–1990 and 2003–2012, respectively, with the measured volume of sediment transported into the reservoir decreasing faster than expected.
2. The variation in the water level, water discharge, and sediment concentration calculated using the model is consistent with that of the measured results, and the calculated cumulative sedimentation volume and sedimentation distribution are basically in line with the measured results, which suggests that the verification results of the model are characterized by good consistency and reliability.
3. Under the water–sediment conditions during 1961–1970 and 1991–2000, the model predicted the estimates of 320 and 430 years for the TGR to reach a sedimentation balance, respectively. Under the new water–sediment conditions, it takes 560 years at most and 450 years at least to reach the sedimentation balance for the TGR, and the corresponding condition is the typical year with less water-less sediment and more water-more sediment, respectively. The findings of this study could serve as a new reference for the long-term safe operation and optimized scheduling of the TGR.
